# INTERPERSONAL DYSFUNCTION PREDICTS SUBSEQUENT FINANCIAL EXPLOITATION VULNERABILITY IN OLDER ADULTS

**DOI:** 10.1093/geroni/igac059.2396

**Published:** 2022-12-20

**Authors:** Duke Han, Aaron Lim, Laura Mosqueda, Annie Nguyen, Tyler Mason, Gali Weissberger, Laura Fenton, Peter Lichtenberg

**Affiliations:** University of Southern California, Alhambra, California, United States; University of Southern California, Alhambra, California, United States; University of Southern California, Alhambra, California, United States; University of Southern California, Alhambra, California, United States; University of Southern California, Los Angeles, California, United States; Bar Ilan University, Ramat Gan, HaMerkaz, Israel; University of Southern California, Alhambra, California, United States; Wayne State University, Detroit, Michigan, United States

## Abstract

The goal of this study was to test whether interpersonal dysfunction, characterized by loneliness and/or dissatisfaction with relationships, is an imminent predictor of financial exploitation vulnerability (FEV) among older adults within a 6-month observation period. This study also tests whether FEV prospectively predicts interpersonal dysfunction. Twenty-six adults aged 50 or older completed a study involving baseline data collection and 13 follow-ups over 6 months. Linear mixed models were used for primary analyses. After adjustment for demographic, psychological, and cognitive covariates, there were between-person effects of FEV and interpersonal dysfunction across follow-ups, suggesting that those with generally higher interpersonal dysfunction compared to other participants also reported greater FEV (B(SE)=1.09(.33), p=.003). There was a within-person effect (B(SE)=.08(.03), p=.007) of elevated interpersonal dysfunction predicting greater FEV two weeks later across all follow-ups. Within-person effect of FEV was not predictive of interpersonal dysfunction (B(SE)=.25(.15), p=.10). Among older adults, individuals with higher interpersonal dysfunction relative to others in the study reported greater FEV throughout the 6-month observation period. Increased loneliness and social dissatisfaction, relative to one’s average level, predicts subsequent increases in FEV, and may be an imminent risk factor for exploitation.

